# Complete Genome Sequence of KPC-Producing *Klebsiella pneumoniae* Strain CAV1193

**DOI:** 10.1128/genomeA.01649-15

**Published:** 2016-01-28

**Authors:** Anna E. Sheppard, Nicole Stoesser, Robert Sebra, Andrew Kasarskis, Gintaras Deikus, Luke Anson, A. Sarah Walker, Tim E. Peto, Derrick W. Crook, Amy J. Mathers

**Affiliations:** a Modernizing Medical Microbiology Consortium, Nuffield Department of Clinical Medicine, John Radcliffe Hospital, Oxford University, Oxford, United Kingdom; b Icahn Institute and Department of Genetics and Genomic Sciences, Icahn School of Medicine, Mount Sinai, New York, New York, USA; c Division of Infectious Diseases and International Health, Department of Medicine, University of Virginia Health System, Charlottesville, Virginia, USA; d Clinical Microbiology, Department of Pathology, University of Virginia Health System, Charlottesville, Virginia, USA

## Abstract

Carbapenem resistance in *Klebsiella pneumoniae*, frequently conferred by the *bla*_KPC_ gene, is a major public health threat. We sequenced a *bla*_KPC_-containing strain of *K. pneumoniae* belonging to the emergent lineage ST941, in order to better understand the evolution of *bla*_KPC_ within this species.

## GENOME ANNOUNCEMENT

*Klebsiella pneumoniae* carbapenemase (KPC) confers multidrug resistance, with KPC-producing *Enterobacteriaceae* becoming increasingly prevalent in nosocomial infections. The most commonly described lineage associated with KPC is *K. pneumoniae* multilocus sequence type ST258, which is found globally (1–4). We previously characterized 37 KPC-producing *K. pneumoniae* isolates from a single health care institution, revealing a high level of genetic diversity, with the most prevalent lineage being ST941 (5). Here, we describe the complete genome sequence of CAV1193, a KPC-producing ST941 isolate from this institution.

CAV1193 was isolated from the ventilated airway of a 46-year-old female on hospital day 31 following a severe trauma. A genetically similar isolate (CAV1199) had been isolated from her sputum on hospital day seven. The patient was given 14 days of intravenous trimethoprim-sulfamethoxazole to which CAV1199 was susceptible (MIC of 40 µg/mL, VITEK2). CAV1193 was isolated 24 days later and was phenotypically resistant to trimethoprim-sulfamethoxazole. The tigecycline zone size had also increased from 18 mm to 21 mm, but otherwise CAV1199 and CAV1193 had identical susceptibility patterns.

PacBio sequencing of CAV1193 and initial *de novo* assembly were performed as previously described (5). To refine the assembly, we also performed Illumina sequencing, as previously described (5). Illumina reads were then mapped to the PacBio assembly using bwa-mem version 0.7.5a-r405 (6) and visualized using Gap5 version 1.2.14-r (7). Unmapped reads were *de novo* assembled using a5-miseq version 20140401 (8) to identify small plasmids, as these were expected to be absent from the PacBio assembly due to size selection of the input DNA (i.e., <7-kb DNA fragments were removed). Plasmid and chromosome structures were closed by resolving repeats at the ends of contigs. As the repeats were generally imperfect, mapping information was used to determine the correct sequence in each case. To validate the final assembly structure, each replicon was relinearized in a nonrepeat region, the Illumina reads were remapped, and Gap5 was used for visualization, confirming the absence of misassemblies. The consensus sequence from mapping was also used to correct minor errors in the assembly sequence (generally single-base indels in homopolymeric regions).

The initial PacBio assembly consisted of five contigs (one chromosomal, four plasmid), with Illumina sequencing identifying one additional small plasmid. The final closed assembly consisted of a chromosome of 5,319,154 bp and five plasmids of 3,741 bp, 49,565 bp, 77,808 bp, 166,486 bp, and 257,944 bp.

The *bla*_KPC_ gene was located on the 49,565-bp plasmid, named pKPC_CAV1193, which was related to the *bla*_KPC_ plasmid pKPC_UVA01, previously identified from another *K. pneumoniae* isolate at the same institution (5). pKPC_CAV1193 differs from pKPC_UVA01 by one single-nucleotide variant and two insertions of 4,878 bp and 1,066 bp, which interrupt genes encoding a resolvase and the conjugal transfer protein TrbI, respectively.

### Nucleotide sequence accession numbers.

The complete genome sequence of CAV1193 has been deposited in GenBank under the accession numbers CP013321 to CP013326.
